# Median Cubital Vein Traveling Deep to the Bicipital Aponeurosis: Anatomical Description With Application to Venipuncture and Vascular Access in the Cubital Fossa

**DOI:** 10.7759/cureus.25105

**Published:** 2022-05-18

**Authors:** Emma Newton, Joe Iwanaga, Aaron S Dumont, R. Shane Tubbs

**Affiliations:** 1 Department of Neurosurgery, Tulane University School of Medicine, New Orleans, USA; 2 Neurosurgery and Ochsner Neuroscience Institute, Ochsner Health System, New Orleans, USA

**Keywords:** complications, anatomy, variation, venous, upper limb

## Abstract

Knowledge of anatomical variations can aid the clinical practitioner in avoiding iatrogenic injury during invasive procedures. Here, we present an unusual finding of the median cubital vein and its relationship with bicipital aponeurosis. This case and relevant reports from the literature are reviewed. Physicians or other health care providers who perform procedures in the cubital fossa, such as venipuncture or vascular access, should be aware of such an anatomical variation.

## Introduction

The cubital fossa is an important anatomical landmark that acts as a transition between the arm and the forearm. Its roof consists of deep fascia and is reinforced by the bicipital aponeurosis (lacertus fibrosus), on top of which typically lies the median cubital vein [1]. The bicipital aponeurosis is a tendon-like structure that emerges from the tendon of the biceps brachii and acts as protection for the underlying median nerve and brachial and ulnar arteries. The median cubital vein (MCV) typically runs superficial to the aponeurosis [2]. The MCV stems proximally from the deep median vein and extends distally to become venous tributaries in the forearm. The cubital fossa is clinically relevant, as it is commonly used for arterial cannulation and venipuncture, and sometimes veins in this area are used for direct heart catheterization or hemodialysis in patients with kidney disease [3]. The MCV is frequently utilized to draw blood, and chemotherapy can also be administered here if the patient in question does not have a port or central venous line [4]. Interestingly, if the patient is unable to tolerate a forearm arteriovenous fistula (AVF), a brachiocephalic AVF can be made with the median cubital vein [5]. The cubital fossa is not only relevant for the purposes of diagnostic testing and treatment, but several pathologies can also arise due to issues in this area. Cubital tunnel syndrome, in which the ulnar nerve becomes compressed or irritated as it runs along the medial epicondyle, can cause significant numbness, tingling, and possible weakness and/or nerve damage. Furthermore, supracondylar fractures of the humerus can interfere with blood supply to the forearm. If left untreated, this can cause Volkmann’s ischemic contracture in which the loss of blood supply causes a deformity of the fingers, wrist, and hand [2].

## Case presentation

**Figure 1:**
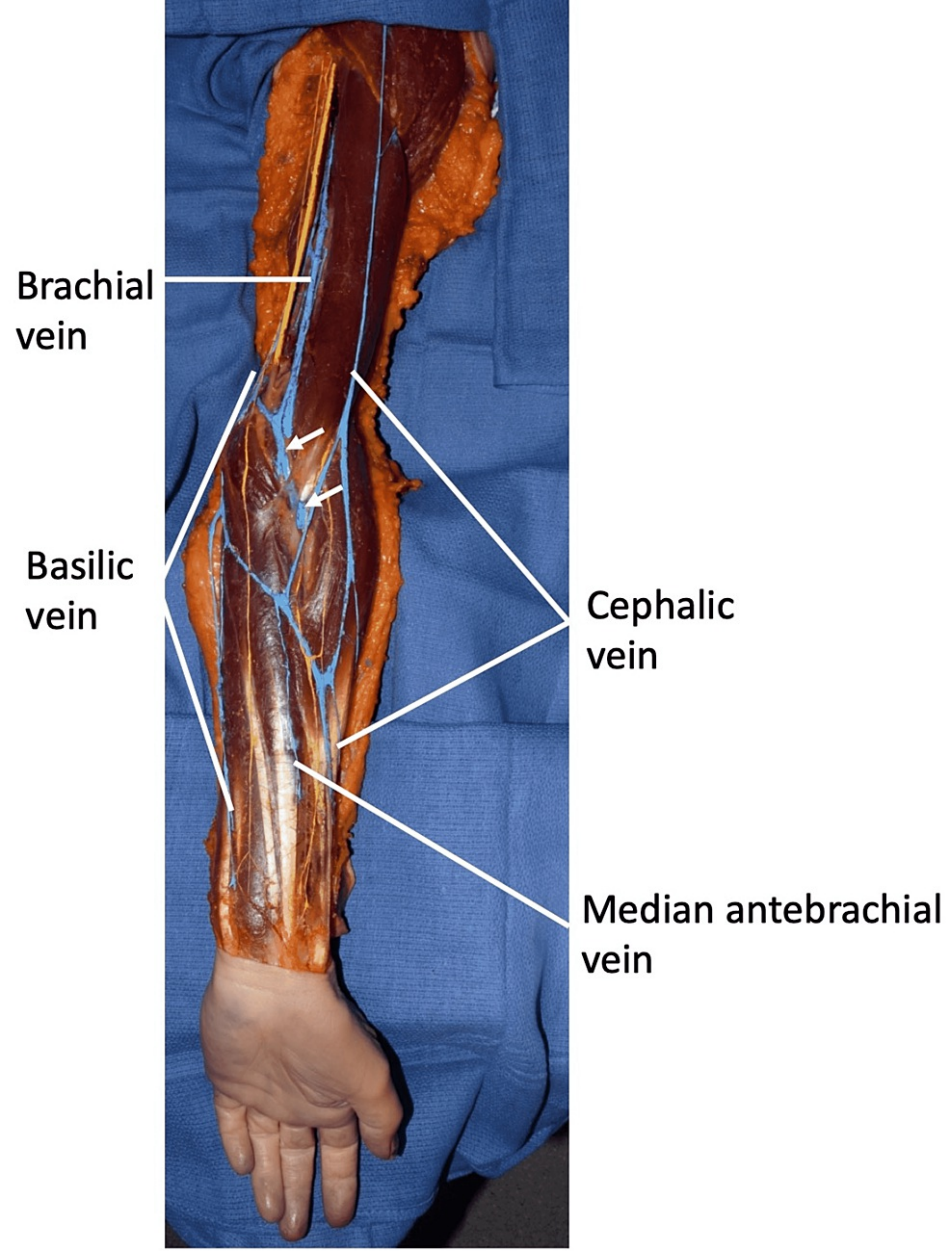
Left upper limb of the cadaver The skin of the anterior arm and forearm has been removed and the superficial veins colored blue for clarity. Note the course of the regional superficial veins and the median cubital vein (arrows) traveling deep to the bicipital aponeurosis.

**Figure 2:**
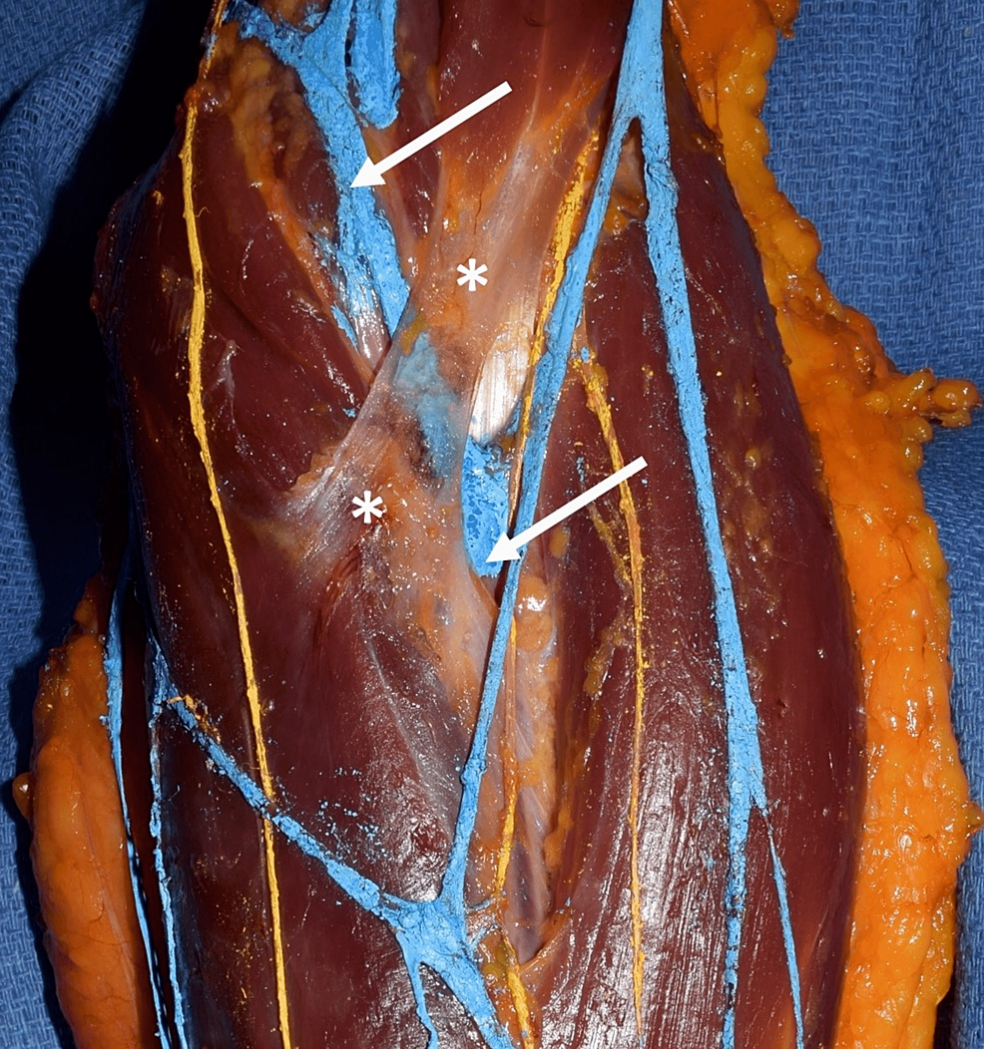
Zoomed-in view of Figure 1 showing the median cubital vein (arrows) traveling deep to the bicipital aponeurosis (*)

During the routine dissection of the upper limb in an 83-year-old at-death male cadaver, an unusual finding was noted in the cubital fossa. The specimen was fresh frozen without evidence of prior surgical scars along the areas dissected. This specimen was found to have a median cubital vein that traveled not superficial to but rather deep to the bicipital aponeurosis (Figures 1-2).

Traveling proximally, the cephalic vein was noted to bifurcate at approximately the midportion of the forearm. The medial part of this bifurcation (medial basilic vein) continued to join the basilic vein approximately 4 cm distal to the medial epicondyle. The lateral part of this bifurcation traveled proximally to join the median cubital vein and then continued more proximally as an additional tributary of the cephalic vein. The median cubital vein ascended to join the basilic vein just proximal to the elbow and deep to the medial antebrachial cutaneous nerve and then continued as the brachial vein. The cephalic vein continued proximally into the deltopectoral triangle and then entered the axillary vein. With elbow extension, the part of the median cubital vein traveling deep to the bicipital aponeurosis was observed to be compressed. This portion under the aponeurosis was approximately 1 cm in width. Flexion of the elbow relieved this compression. The median antecubital vein was small and drained into the medial-most part of the bifurcated cephalic vein. No additional musculoskeletal or neurovascular anatomical variations were noted in the left upper limb and no similar findings were observed in the contralateral upper limb. 

## Discussion

Due to the clinical significance of this area (e.g., venipuncture, vascular access of the cubital fossa), it is important to note the subtypes in which the structures, specifically the veins, can be arranged. In Type I (N type) superficial veins, the cephalic and basilic veins join in the cubital fossa. This subtype has been found to be the most common in several studies [1,3,6]. In Type II (M type), the median cubital vein connects the basilic and cephalic veins. In Type III (I or O type), the brachial cephalic vein is either missing or poorly developed in the cubital region. In Type IV, there is no communicating branch between the basilic and cephalic veins [2]. In the Type V subtype, the cephalic and basilic veins connect by an arc proximal to the cubital fossa. In Type VI, the basilic and cephalic veins are connected by two separate median cubital veins, and in Type VII, the cephalic vein is divided into two branches that become the basilic vein and an accessory cephalic vein [3]. Yammine and Erić also described a Type VIII pattern with a median antebrachial vein of the forearm splitting into the medial basilic and median cubital veins and with a duplicated proximal cephalic vein [7]. Our specimen was found to not fit any of these previously noted types and, thus, might be considered a Type IX pattern of superficial cubital veins.

Variations in the typical anatomy of this site should be noted due to this site’s clinical importance and the potential for procedural complications and neurovascular compression. Several variations have been previously reported. In one case study, the cephalic vein bifurcated to form the cephalic vein proper and the median cubital vein in the cubital fossa. The MCV in this case passed deep to two slips of the bicipital aponeurosis, which is unusual, as it is typically superficial [8]. Another case report detailed a bifurcated aponeurosis that split into a medial and lateral slip [9]. For our case, in elbow extension, the common positioning for vascular access procedures of the cubital fossa, the median cubital vein would probably not be seen due to compression by the overlying bicipital aponeurosis. Therefore, venipuncture of the median cubital vein at this location would be difficult. If the brachial artery were targeted in our specimen, not visualizing the more superficially located median cubital vein might lead to excessive hemorrhage when approaching the artery, as the vein would not be apparent.

## Conclusions

In this case report, we presented a case in which the median cubital vein runs deep to the bicipital aponeurosis. There are several previously documented anatomical variants possible in this area, and it is likely that more will be discovered. Physicians should be aware of variants in the cubital fossa such as this, especially during venipuncture or any procedure involving vascular access. Ignorance of potential variants in this could lead to potential complications, including but not limited to reduction in blood supply to the hand and forearm, cubital tunnel syndrome, and ischemic contracture.

## References

[1] Ellis H, Feldman S, Harrup-Griffiths W (2010). The clinical anatomy of the antecubital fossa. Br J Hosp Med (Lond).

[2] Bains KNS, Lappin SL (2022). Anatomy, Shoulder and Upper Limb, Elbow Cubital Fossa. StatPearls. StatPearls Publishing.

[3] Ghasem GM, Nasiri K, Sagha M (2021). Variation of superficial veins of cubital fossa among students of Ardabil University of Medical Sciences. Transl Res Anat.

[4] Yoshida Y, Hoshino S, Aisu N (2015). Administration of chemotherapy via the median cubital vein without implantable central venous access ports: port-free chemotherapy for metastatic colorectal cancer patients. Int J Clin Oncol.

[5] Guifo ML, Teuwafeu DG, Bwelle MG, Bang GA, Chichom MA, Ndoumbe A, Essomba AG (2018). Brachiocephalic A-V fistula through the median cubital vein; a reliable option to failure of forearm fistulas. A case report from CHU Yaoundé. Int J Surg Case Rep.

[6] del Sol M, Lillo E, Lobos L, Vasquez B (2012). Study of the veins of the cubital fossa through helical computed tomography and its clinical application. Int J Morphol.

[7] Yammine K, Erić M (2017). Patterns of the superficial veins of the cubital fossa: a meta-analysis. Phlebology.

[8] Bhat N, Bhat KM, D'Souza AS, Kotian SR (2017). Additional muscle slip of bicipital aponeurosis and its anomalous relationship with the median cubital vein. Sultan Qaboos Univ Med J.

[9] Nayak SB, Swamy RS, Shetty P, Maloor PA, Dsouza MR (2016). Bifurcated bicipital aponeurosis giving origin to flexor and extensor muscles of the forearm - a case report. J Clin Diagn Res.

